# Complete Digital Disconnection as a Prognostic Marker of Mortality or Residential-Care Transition in Older Adults With Cardiovascular Risk: A Nationally Representative Cohort Study

**DOI:** 10.21203/rs.3.rs-9873742/v1

**Published:** 2026-06-22

**Authors:** Peng Liu, Ruilong Gao, Yaliu Yang, Zhengqin Zhai, Yifeng Zhou

**Affiliations:** China-Japan Friendship Hospital; China-Japan Friendship Hospital; China-Japan Friendship Hospital; China-Japan Friendship Hospital; China-Japan Friendship Hospital

**Keywords:** digital divide, internet use, telehealth, aged, cardiovascular diseases, mortality, residential care, health equity, cohort studies, National Health and Aging Trends Study

## Abstract

**Background:**

Routine health care increasingly requires digital access for appointment scheduling, medication refills, test-result review, clinician messaging, remote monitoring, and telehealth. For older adults with cardiovascular-risk conditions, complete internet disconnection may indicate accumulated geriatric vulnerability and barriers to continuous care. We examined whether complete digital disconnection was associated with mortality or residential-care transition and whether the risk gradient was driven by connectivity rather than telehealth use.

**Methods:**

We constructed staggered-entry prospective cohorts using rounds 11–14 of the National Health and Aging Trends Study (2021–2025). Community-dwelling Medicare beneficiaries aged ≥65 years with hypertension, heart disease, or diabetes entered the cohort in round 11, 12, or 13 and were followed through round 14. Digital integration was categorized as telehealth use, connected non-use, or complete disconnection. Outcomes were all-cause mortality and a composite of death or transition to nursing-home or residential care. The primary analysis used survey-weighted discrete-time survival models with entry-cohort fixed effects and participant-level cluster-robust variance estimation. Models were sequentially adjusted for demographic, socioeconomic, health, frailty-related, and geriatric-vulnerability factors. Robustness was assessed using multiple imputation, competing-risks models, E-values, and sensitivity analyses addressing reverse causation.

**Results:**

Among 12,139 person-baseline observations from 6,530 adults, the survey-weighted prevalence of complete disconnection was 29.7%. Compared with non-disconnected participants, completely disconnected adults had a higher unadjusted risk of the composite outcome; after adjustment for health and frailty-related factors, the association attenuated but persisted (composite hazard ratio [HR], 1.39; 95% CI, 1.12–1.73; mortality HR, 1.50; 95% CI, 1.16–1.94). The survey-weighted 3-year absolute risk difference was 12.1 percentage points. Telehealth users and connected non-users had similar adjusted risks (composite HR, 0.99; 95% CI, 0.79–1.24). Associations weakened after adjustment for functional status and exclusion of first-interval events.

**Conclusions:**

Complete digital disconnection was a reproducible and readily measured prognostic marker of mortality or residential-care transition among older adults with cardiovascular-risk conditions. These findings support a prognostic rather than causal interpretation. Age-friendly digital care should preserve offline-accessible pathways for older adults who remain completely disconnected.

## Background

The COVID-19 pandemic led to an abrupt expansion of telehealth, but its most durable legacy may be the broader reorganization of routine care around a “digital-default” model: appointment scheduling, prescription refills, laboratory-result review, secure clinician messaging, remote-monitoring enrollment, and, increasingly, clinical visits themselves are expected to occur through digital channels [1, 2]. Patient-facing digital access has therefore become a gateway to care rather than an ancillary convenience. The U.S. public health emergency ended on May 11, 2023, but the digital infrastructure established during the pandemic has persisted; after a brief lapse, Medicare telehealth flexibilities were extended under the Consolidated Appropriations Act, 2026, through December 31, 2027 [3, 4].For older people who account for a large share of cardiovascular care, digital access is thus shifting from a convenience to a structural condition for entering and remaining engaged in the health system, a concern increasingly conceptualized as a “digital determinant of health [5–7].”

This shift is especially consequential for conditions that depend on continuity of care. Cardiovascular management is unusually continuity-dependent: blood pressure and glucose levels require periodic monitoring and medication titration, anticoagulation requires regular follow-up, and remote and self-measured blood-pressure monitoring are becoming standard portal- and app-mediated components of care [8]. Accordingly, disruption of these continuous management chains through digital disconnection may manifest more directly and rapidly in cardiovascular populations than in many other chronic-disease groups; these populations are also event-rich, providing the statistical power needed to study hard end points. Social disconnection, of which digital disconnection is an increasingly salient form, is itself an established correlate of cardiovascular morbidity and mortality [9, 10].

Across the Health and Retirement Study, the English Longitudinal Study of Ageing, the Survey of Health, Ageing and Retirement in Europe, and other aging cohorts, internet non-use has already been associated with mortality, depression, frailty, functional dependence, and cognitive decline among older adults [11–15]. The contribution of the present study is therefore not simply to show that being offline is associated with worse outcomes, but to make three specific advances. First, the study is situated explicitly in the post-pandemic digital-default environment, in which disconnection increasingly means not merely lacking a convenience but being excluded from a primary entry point to care. Second, it introduces a connected non-user comparison group, enabling digital connection to be distinguished from telehealth adoption—a distinction that the digital-equity literature, which has largely focused on increasing telehealth uptake, has not tested against hard outcomes [5, 16]. Third, it examines death and loss of independent living rather than intermediate or self-reported outcomes.

Against this background, we addressed two questions. First, does complete digital disconnection remain associated with a higher risk of death or loss of independent living after full adjustment for demographic, socioeconomic, health/frailty, and functional factors? Second, is the risk gradient across the digital-integration continuum characterized by a discontinuity at complete disconnection rather than by a smooth dose-response relationship with telehealth adoption? We prespecified that the central claim would be framed in terms of a prognostic marker, with any structural-barrier interpretation presented only as a hypothesis; accordingly, we used disciplined terminology throughout—“marker,” “associated with,” and “consistent with”—and avoided causal language.

## Methods

### Study Design and Data Source

**Figure 1:**
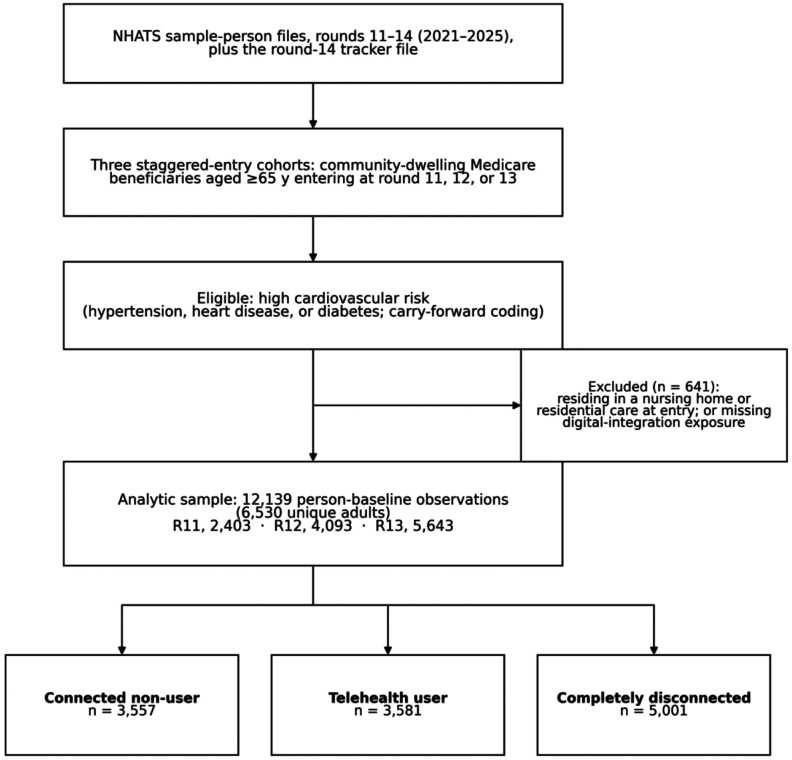
Study flow diagram. Construction of the three staggered-entry cohorts from NHATS rounds 11–14, eligibility and exclusion criteria, and the analytic sample.

We conducted a nationally representative, staggered-entry prospective cohort study using public-use sample-person files from NHATS rounds 11–14 and the round 14 tracker file. NHATS is an ongoing panel study of Medicare beneficiaries aged ≥ 65 years, initiated in 2011 using multistage area-probability sampling with periodic replenishment and oversampling of older age groups and Black beneficiaries [17]. Three entry cohorts were defined: participants who met eligibility criteria at round 11 (2021), round 12 (2022), or round 13 (2023), each of whom was followed through round 14 (2025). The study flow, including eligibility criteria, exclusions, and analytic sample sizes, is shown in Fig. 1. Reporting followed the STROBE statement and the RECORD extension [18, 19].

### Analytic Population and Unit of Analysis

The analytic population comprised community-dwelling older adults at high cardiovascular risk, defined by a reported diagnosis of heart disease, hypertension, or diabetes in the NHATS chronic-condition module. Because these items carry forward prior reports, values indicating either newly reported or previously reported diagnoses were counted as present; restricting the definition to newly reported diagnoses alone would have omitted approximately half of prevalent cases. Participants already residing in a nursing home or residential-care setting at entry were excluded from the residential-care risk set. An established cardiovascular disease covariate, defined as prior myocardial infarction, heart disease, or stroke, was constructed using only data accrued up to and including the entry round to prevent look-ahead and immortal-time bias.

The primary analysis used a stacked person-period structure across the three entry cohorts. Because the same individual could meet eligibility criteria in more than one entry round, observations were not fully independent. We therefore did not treat the three cohorts as independent replications, and the primary model used participant-level cluster-robust variance estimation, as described below. The headline sample size of 12,139 refers to person-baseline observations: round 11, n = 2,403; round 12, n = 4,093; and round 13, n = 5,643. These observations corresponded to 6,530 unique individuals; descriptive tables report unweighted person-level counts.

### Exposure: The Digital-Integration Gradient

The exposure was derived solely from two technology-module items—whether the participant went online and whether the participant had an internet telehealth visit with a medical provider—to avoid cross-module inconsistency. Three mutually exclusive exposure levels were defined: telehealth users, defined as those who were online and reported an internet telehealth visit; connected non-users, defined as those who were online but reported no such visit; and completely disconnected participants, defined as those who were offline. The primary contrast compared offline participants with non-disconnected participants; the secondary contrast compared telehealth users with connected non-users. Although connectivity is intrinsically time-varying, the primary analysis treated connectivity as a time-fixed baseline exposure measured at the entry round.

### Outcomes and Covariates

The two co-primary outcomes were all-cause mortality, ascertained from tracker case-status codes, and a composite hard end point of death or transition to nursing home or residential care, ascertained from the residential-status variable. Because the composite end point was numerically dominated by death, mortality and residential-care transition were also analyzed separately, with residential-care transition additionally analyzed in a competing-risk framework.

Covariates were grouped into four adjustment blocks: demographic factors, M1, including age category, sex, race/ethnicity, and education; socioeconomic factors, M2, including Medicaid coverage, living alone, and marital status; health/frailty factors, M3, including comorbidity count, self-rated health, dementia, and established cardiovascular disease; and geriatric vulnerability factors, M4, including help with activities of daily living, namely eating, bathing, toileting, and dressing, frequency of going outside, depression screen based on the Patient Health Questionnaire-2, self-rated memory, and household-income tertile. All covariates were measured at the entry round. M3 was prespecified as the primary adjustment level. M4 was added because digital disconnection is strongly collinear with functional status, and an association that persists after functional adjustment is more informative as a prognostic marker.

### Statistical Analysis

All analyses used round-specific NHATS analytic weights. Baseline characteristics are reported as unweighted counts and survey-weighted percentages. The primary analytic model was a survey-weighted discrete-time survival model with a complementary log-log link, fitted to the person-period data structure, which yields interpretable hazard ratios for each outcome [20, 21]. To account for non-independence across the stacked cohorts, the primary stacked model was fitted to the pooled person-period dataset using round-specific analytic weights, entry-cohort fixed effects, and participant-level cluster-robust variance estimation to account for repeated contributions by the same participant. This model is reported as the primary result. As a consistency check, each entry cohort was additionally estimated separately with incorporation of the NHATS strata and primary sampling units; cohort-specific estimates were then combined using inverse-variance random-effects meta-analysis, with heterogeneity summarized by I^2^ [22]. Adjustment proceeded stepwise: M0 included structural terms only, followed by M1 through M4 as defined above.

For the secondary contrast, we directly compared telehealth users with connected non-users. Because no equivalence margin was prespecified, we report this analysis as a comparison and do not claim formal statistical equivalence.

To assess robustness, we performed six sensitivity and bias analyses. First, we computed E-values, defined as the minimum strength of association on the risk-ratio scale that an unmeasured confounder would need to have with both the exposure and the outcome to explain away the observed association [23]. Second, we re-estimated the primary model using multiple imputation by chained equations, with m = 10 imputations, for the 1%–2% covariate missingness, rather than relying solely on complete cases [24, 25]. Third, we analyzed residential-care transition as a competing risk using both a cause-specific discrete-time model and a Fine–Gray subdistribution model, with death as the competing event [26]. Fourth, we excluded events in the first follow-up interval after entry to guard against reverse causation. Fifth, we restricted the analysis to self-respondents to address proxy-response entanglement. Sixth, we characterized loss to follow-up by exposure group and bounded its potential effect using extreme-scenario analyses. Analyses were conducted using R, version 4.5.2.

## Results

### Baseline Characteristics

Among 12,139 person-baseline observations, corresponding to 6,530 unique adults, 5,001 observations, or 41.2% of unweighted observations, were contributed by completely disconnected adults. The survey-weighted population prevalence of complete digital disconnection was 29.7%. The three exposure groups differed systematically (Table 1). Compared with the connected groups, the offline group was older, was more often from racially and ethnically minoritized groups, had lower educational attainment and household income, was more likely to have Medicaid coverage, had a higher burden of comorbidity, dementia, and depression, reported worse self-rated health and memory, was more often limited in activities of daily living and in going outside, and was more likely to require a proxy respondent. Among completely disconnected adults, 10.7% needed help with two or more activities of daily living and 20.2% screened positive for depression, compared with 3.9% and 10.8%, respectively, among connected non-users. This confounding structure—the greater vulnerability of the offline group across nearly all measured domains—was the central interpretive challenge and is therefore emphasized throughout the analyses.

### Event Counts and Absolute Risk

During follow-up, 1,373 composite events occurred, including 1,056 deaths and 353 transitions to nursing home or residential care. The composite event proportion was 15.4% among offline participants, 9.1% among connected non-users, and 7.8% among telehealth users (771 of 5,001, 323 of 3,557, and 279 of 3,581, respectively; Table 2). On the absolute scale, the weighted 3-year cumulative incidence of the composite end point in the longest-followed cohort, round 11, was 25.4% among offline participants and 12.9% among connected non-users. The survey-weighted absolute risk difference between offline and non-disconnected adults was 4.9 percentage points at 1 year, 8.6 percentage points at 2 years, and 12.1 percentage points at 3 years, with corresponding 95% CIs of 3.8–5.9, 6.4–10.8, and 8.5–15.7. The cumulative-incidence comparison contrasts offline participants specifically with connected non-users, whereas the Panel B risk differences compare offline participants with all non-disconnected adults.

### Disconnection and the Hard End Points

**Figure 2.**
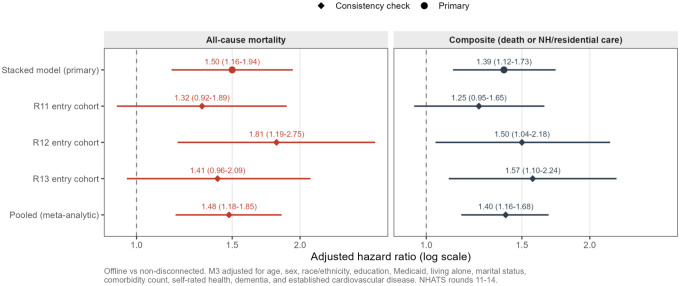
Complete digital disconnection and hard end points (fully adjusted, M3) Stacked model = primary result; per-cohort and meta-analytic estimates shown as a consistency check Fully adjusted (M3) hazard ratios for complete digital disconnection (offline vs non-disconnected). The stacked model is the primary result; per-cohort and meta-analytic estimates are shown as consistency checks. NHATS rounds 11–14.

**Figure 3.**
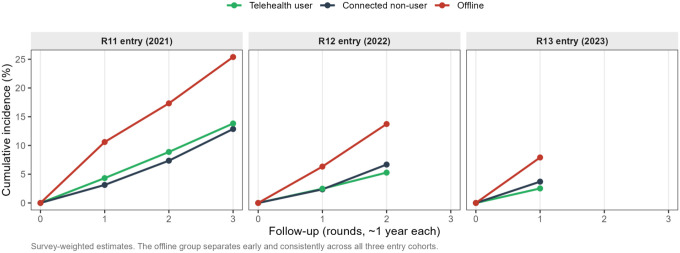
Weighted cumulative incidence of the composite end point Death or transition to nursing home/residential care, by digital-integration group Survey-weighted cumulative incidence of the composite end point, death or transition to nursing home or residential care, by digital-integration group and entry cohort. The offline group separates early and consistently; follow-up length differs by entry round.

In the primary stacked model, completely disconnected adults had an approximately 2.4-fold higher crude risk of the composite end point than non-disconnected adults (HR, 2.38; 95% CI, 2.00–2.83). Stepwise adjustment produced monotonic attenuation (Fig. 2; Additional file 1, Figure S1). After full health/frailty adjustment (M3), the HR was 1.39 for the composite end point and 1.50 for all-cause mortality, with corresponding 95% CIs of 1.12–1.73 and 1.16–1.94. The per-cohort and meta-analytic estimates, retained as consistency checks, were closely concordant with the stacked results: the meta-analytic HR was 1.40 for the composite end point and 1.48 for mortality, with corresponding 95% CIs of 1.16–1.68 and 1.18–1.85. There was no detectable between-cohort heterogeneity (I^2^ = 0%), suggesting that the choice of pooling method did not materially affect the conclusion (Table 3). The survey-weighted cumulative-incidence curves for the composite end point separated early between the offline group and the two connected groups, with a consistent pattern across all three entry cohorts (Fig. 3).

### The Decisive Divide Is Connectivity, Not Telehealth Adoption

**Figure 4.**
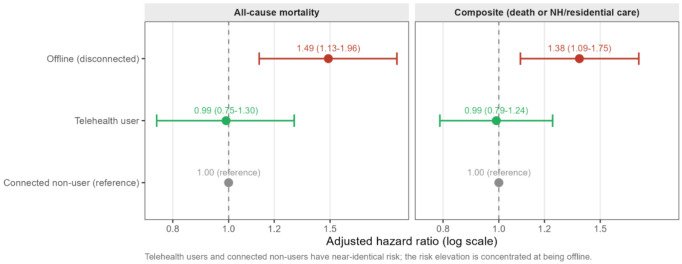
The decisive divide is connectivity, not telehealth adoption Fully adjusted (M3) stacked-model hazard ratios across the digital-integration gradient Fully adjusted (M3) stacked-model hazard ratios across the three-level digital-integration gradient, with connected non-users as the reference. Telehealth users and connected non-users have near-identical risk; the elevation is concentrated at complete disconnection.

In the stacked model, telehealth users and connected non-users had nearly identical fully adjusted risks: the HR was 0.99 for both the composite end point and mortality, with corresponding 95% CIs of 0.79–1.24 and 0.75–1.30. Both connected groups had substantially lower risk than the offline group (Fig. 4). The single stacked estimate also addressed an interpretive limitation of cohort-by-cohort pooling: the per-cohort estimates for this secondary contrast were heterogeneous (I^2^ = 61%, ranging from 0.73 to 1.23 across rounds). Therefore, the pooled meta-analytic estimate should be interpreted cautiously, whereas the stacked estimate of approximately 1.0 provides the more stable summary. The risk gradient was therefore characterized by a discontinuity at complete disconnection rather than by a smooth decline with increasing telehealth uptake. This interpretation has an important boundary: telehealth users and connected non-users both fall on the “online” side of the exposure gradient, a status positively selected by health and socioeconomic factors. Thus, this analysis characterizes the shape of the gradient—specifically, its discontinuity at being offline—rather than establishing the causal status of connectivity itself.

### Adjustment for Functional Status

Because the offline group had substantially greater functional impairment, we added the geriatric-vulnerability block (M4). Additional adjustment for help with activities of daily living, frequency of going outside, depression, self-rated memory, and income further attenuated the association. The composite HR decreased from 1.39 in M3 to 1.25 in M4 (95% CI, 1.00–1.56), and the mortality HR decreased from 1.50 to 1.33 (95% CI, 1.03–1.73) (Table 3; Figure S1). The direction of association was preserved, and the mortality estimate remained statistically significant; however, the composite estimate became borderline, suggesting that a substantial portion of the crude association was accounted for by measurable functional decline. This pattern is consistent with a prognostic-marker interpretation and argues against a strong independent causal effect.

### Residential-Care Transition as a Competing Risk

Because the composite end point was dominated by death, we analyzed transition to nursing home or residential care separately, treating death as a competing event. The fully adjusted association between complete disconnection and residential-care transition was positive but not statistically significant: the cause-specific HR was 1.24 (95% CI, 0.84–1.82), and the Fine–Gray subdistribution HR was 1.18 (95% CI, 0.80–1.74). This pattern likely reflects both the smaller number of residential-care transitions, n = 353, and the concentration of the composite signal in mortality.

### Unmeasured Confounding, Reverse Causation, and Missing Data

Several analyses informed the causal interpretation (Table 4). E-values were moderate. For the fully adjusted M3 estimates, an unmeasured confounder would need to be associated with both the exposure and the outcome by risk ratios of approximately 2.12 for the composite end point and 2.36 for mortality to fully explain the observed associations; the corresponding values for the confidence limits were 1.47 and 1.58. For the functionally adjusted M4 composite estimate, the E-value decreased to 1.80, with a value of 1.06 for the confidence limit. Multiple imputation for covariate missingness reproduced the complete-case results, with an HR of 1.40 for the composite end point and 1.53 for mortality.

Two analyses materially weakened a causal interpretation. Excluding first-interval events moved the stacked composite estimate to 1.21 (95% CI, 0.88–1.66) and attenuated the estimate in the longest-followed cohort to the null (round 11 HR, 1.01), consistent with possible reverse causation in which health decline near the end of life precipitates withdrawal from connectivity. Restricting the analysis to self-respondents also shifted estimates toward the null. Loss to follow-up was modestly higher in the offline group than among connected non-users, 14.0% versus 11.3%. Under extreme-scenario bounding, the composite HR ranged from 1.07 to 1.85. Taken together, the moderate E-values and the sensitivity-analysis findings support interpreting the association as prognostic rather than causal.

## Discussion

### Principal Findings

In this nationally representative post-pandemic cohort of older adults at high cardiovascular risk, complete digital disconnection was common, with a survey-weighted prevalence of 29.7%, and was associated with an approximately 1.4- to 1.5-fold higher risk of death or loss of independent living after adjustment for demographic, socioeconomic, and health/frailty factors. The direction of association was consistent across three staggered-entry cohorts and under multiple imputation. The main distinction was connectivity rather than telehealth adoption: telehealth users and connected non-users had nearly identical risks, whereas the excess risk was concentrated among participants who were offline. The central message is that, in the post-pandemic digital-default care environment, complete internet disconnection identifies a subgroup of older adults at high cardiovascular risk who are more likely to die or lose independent living; however, this association should be interpreted as a prognostic marker rather than as evidence of a causal effect.

### A Prognostic Marker, Not a Causal Factor

We do not claim that digital disconnection causes death, and four lines of evidence warrant caution. First, the crude approximately 2.4- to 2.7-fold association was substantially attenuated after adjustment, and further adjustment for functional status reduced the composite estimate to a borderline value of 1.25, suggesting that much of the association was accounted for by measurable frailty, functional, socioeconomic, and cognitive differences. Second, the E-values were moderate, indicating that residual confounding of moderate strength could account for the remaining association. Third, excluding first-interval events moved the estimate toward the null and attenuated the estimate in the longest-followed cohort to the null; restriction to self-respondents also shifted estimates toward the null. These findings are consistent with possible reverse causation and proxy-response entanglement. Fourth, the marker is, by construction, a single binary item that compresses multidimensional vulnerability into a simplified exposure measure. Digital disconnection is therefore best understood as a marker that is directionally reproducible across entry cohorts but sensitive to reverse-causation and proxy-response analyses. Its potential role as a structural barrier to care remains a hypothesis that would require designs with stronger causal identification, such as natural experiments or instrumental-variable approaches, to test.

### Comparison With Prior Work

These findings are consistent with multi-cohort evidence that internet use is associated with lower mortality and cardiometabolic risk among older adults, as well as with broader literature linking digital exclusion to depression, loneliness, functional dependence, and cognitive decline [11–13, 15]. They also align with evidence that social isolation is associated with cardiovascular events and mortality [27–29]. The contribution of the present study is its post-pandemic, hard-end-point framing and, in particular, the inclusion of a connected non-user comparison group, which helps refine the policy target. Whereas much of the digital-health-equity literature and the Medicare telehealth debate have centered on increasing telehealth adoption and addressing barriers to telehealth readiness, our data suggest that telehealth adoption versus non-adoption was not meaningfully associated with hard outcomes; the more informative distinction was whether an older adult was connected at all [3, 4, 16, 30–34]. This pattern aligns with the conceptualization of digital access as a distinct determinant of health and with evidence that disparities in patient-portal use and digital engagement persist, and in some respects widen, even as overall use increases [7, 30, 31, 35].

### Implications for Digital Health System Design

Regardless of whether the relationship is causal, this marker may have practical value for the design and deployment of patient-facing digital health systems. Before digital cardiovascular programs—such as patient portals, remote monitoring, secure-messaging pathways, and telehealth-first care models—are scaled, a single question about connectivity may help identify a group with an approximately 1.4- to 1.5-fold higher risk. More broadly, our data suggest a potential misalignment in post-pandemic digital-equity priorities: efforts have concentrated on increasing telehealth uptake, yet uptake was only weakly related to hard outcomes, whereas the prognostically important group was the approximately 30% of this population, survey-weighted, who were completely offline. We therefore suggest that digital cardiovascular programs treat offline status as a required implementation variable. Trials, portal rollouts, remote-monitoring programs, and telehealth-first care models should report the proportion of eligible patients who are completely offline and specify non-digital alternatives—such as institutionalized telephone and in-person channels, community digital-access points, and device and literacy support—so that digitizing a care pathway does not systematically disadvantage patients at the highest baseline risk.

### Strengths and Limitations

Strengths of this study include national representativeness, hard end points, a staggered-entry design, a connected non-user comparison group, a primary model that accounts for non-independence across the stacked cohorts, and a prespecified, transparent causal-inference appraisal that includes multiple imputation, competing-risk, and loss-to-follow-up analyses. Several limitations should be noted. Digital disconnection is strongly collinear with frailty, cognition, function, and socioeconomic status. Although the association persisted after functional adjustment, the E-values were only moderate, and residual confounding cannot be excluded. Health decline may precipitate withdrawal from connectivity, and sensitivity analyses suggest that this reverse-causal pathway may have contributed to the observed association. The exposure was measured using a single item at a single time point, whereas connectivity is inherently time-varying. The composite end point was dominated by death, and the residential-care component alone was not statistically significant. Differential loss to follow-up could have introduced informative censoring, although bounding analyses suggested that its effect was limited. Comorbidity and functional measures were self-reported. In addition, because the same individual could contribute to more than one entry cohort, the cohorts were not independent and were not treated as independent replications.

## Conclusions

In a nationally representative post-pandemic population of older adults at high cardiovascular risk, complete digital disconnection was a reproducible and easily measured prognostic marker of death or loss of independent living, with a fully adjusted HR of approximately 1.4 to 1.5. The main divide was connectivity rather than telehealth adoption. Because the magnitude of the association depended on sensitivity analyses, the evidence supports a prognostic rather than causal interpretation. A single connectivity question may support rapid risk stratification before patient-facing digital cardiovascular care is scaled, and digital-health-equity policy should prioritize offline-accessible safety nets for people who are completely disconnected.

## Supplementary Material

Supplementary Files

This is a list of supplementary files associated with this preprint. Click to download.

- Additionalfile1Supplementary.docx

## Figures and Tables

**Table 1:** Baseline characteristics of older adults at high cardiovascular risk, by digital-integration group (NHATS rounds 11–14).

Characteristic	Connected non-user	Telehealth user	Completely disconnected
**Unweighted N = 6530 (1,969 / 1,783 / 2,778)**			
**Age group, y**			
65–69	264 (18.7)	310 (23.8)	266 (13.7)
70–74	487 (33.9)	552 (39.1)	474 (24.5)
75–79	579 (27.4)	450 (21.8)	641 (25.3)
80–84	351 (12.7)	309 (10.8)	636 (18.2)
85–89	186 (5.2)	112 (3.5)	446 (11.6)
>=90	102 (2.1)	50 (1.0)	315 (6.7)
**Sex**			
Male	877 (47.9)	831 (50.2)	1099 (44.5)
Female	1057 (52.1)	931 (49.8)	1595 (55.5)
**Race/ethnicity**			
Non-Hispanic White	1304 (81.5)	1252 (84.9)	996 (54.3)
Non-Hispanic Black	322 (7.3)	270 (6.3)	818 (17.8)
Hispanic	230 (6.3)	175 (4.6)	759 (20.9)
Other/multiple	78 (4.9)	65 (4.3)	121 (7.0)
**Education**			
Above high school	1746 (92.6)	1683 (96.9)	1588 (66.8)
High school or equivalent	119 (4.9)	49 (2.4)	488 (16.6)
Below high school	63 (2.5)	22 (0.7)	595 (16.6)
**Marital status**			
Married/partnered	1002 (55.7)	1063 (65.6)	1025 (40.7)
Not married/partnered	967 (44.3)	718 (34.4)	1750 (59.3)
**Living arrangement**			
Not living alone	1394 (72.5)	1350 (78.3)	1875 (66.8)
Living alone	575 (27.5)	433 (21.7)	903 (33.2)
**Medicaid coverage**			
No	1730 (91.4)	1625 (93.8)	1777 (70.5)
Yes	227 (8.6)	152 (6.2)	906 (29.5)
Comorbidity count (0–10), mean	2.78	2.91	3.21
**Self-rated health**			
Excellent	155 (8.2)	131 (8.8)	144 (5.9)
Very good	630 (34.8)	606 (37.3)	447 (18.1)
Good	787 (39.1)	688 (35.9)	1024 (36.9)
Fair	337 (15.1)	291 (14.1)	902 (30.3)
Poor	59 (2.9)	67 (3.9)	259 (8.8)
**Dementia**			
No	1924 (98.0)	1748 (98.3)	2519 (92.2)
Yes	45 (2.0)	35 (1.7)	259 (7.8)
**Established cardiovascular disease**			
No	1373 (72.1)	1200 (70.0)	1776 (63.3)
Yes	596 (27.9)	583 (30.0)	1002 (36.7)
**Help with activities of daily living**			
None	1754 (90.3)	1579 (90.4)	2121 (78.9)
1 activity	123 (5.8)	124 (6.5)	297 (10.4)
>=2 activities	92 (3.9)	80 (3.1)	360 (10.7)
**Frequency of going outside**			
Most days	1618 (86.2)	1488 (87.5)	1731 (68.8)
Some days	262 (10.6)	208 (9.1)	620 (18.7)
Rarely or never	89 (3.1)	87 (3.4)	426 (12.4)
Unknown	0 (0.0)	0 (0.0)	1 (0.0)
**Depression screen (PHQ-2)**			
Negative	1755 (88.9)	1587 (90.6)	2172 (78.7)
Positive	208 (10.8)	191 (9.1)	582 (20.2)
Unknown	6 (0.3)	5 (0.3)	24 (1.1)
**Self-rated memory**			
Good	731 (41.1)	795 (47.2)	668 (25.9)
Fair	810 (39.8)	698 (38.5)	964 (36.9)
Poor	396 (18.2)	274 (13.8)	919 (31.1)
Unknown	32 (0.9)	16 (0.4)	227 (6.2)
**Household income tertile**			
Middle	572 (29.0)	491 (26.2)	523 (20.9)
Low	365 (15.9)	237 (10.0)	1095 (37.3)
High	541 (31.8)	679 (44.1)	197 (9.0)
Unknown	491 (23.3)	376 (19.6)	963 (32.9)
**Interview type**			
Self-respondent	1939 (99.2)	1767 (99.6)	2552 (93.9)
Proxy respondent	30 (0.8)	16 (0.4)	226 (6.1)

*Note*. Values are unweighted n (survey-weighted %) unless noted. Percentages are weighted to the Medicare population aged > = 65 years. Counts may not sum to totals because of rounding and item missingness. PHQ-2: Patient Health Questionnaire-2.

**Table 2:** Event counts and absolute risk.

Panel A. Event counts by digital-integration group				
Exposure group	Person-baseline obs	Deaths, n (%)	Residential-care transition, n (%)	Composite end point, n (%)
Connected non-user	3,557	227 (6.4)	98 (2.8)	323 (9.1)
Telehealth user	3,581	201 (5.6)	84 (2.3)	279 (7.8)
Completely disconnected	5,001	628 (12.6)	171 (3.4)	771 (15.4)
Total	12,139	1,056 (8.7)	353 (2.9)	1,373 (11.3)
*Note*. Unweighted event counts and proportions. The composite end point is death or transition to nursing home/residential care. Death and residential-care transition were not mutually exclusive; the composite counted each person-baseline once at the first qualifying event.				

Panel B. Survey-weighted absolute risk difference for the composite end point, by follow-up year		
Follow-up	Entry cohorts contributing	Survey-weighted absolute risk difference, percentage points (95% CI)
1 year	3 (R11-R13)	4.9 (3.8 to 5.9)
2 years	2 (R11-R12)	8.6 (6.4 to 10.8)
3 years	1 (R11)	12.1 (8.5 to 15.7)
*Note*. Offline vs non-disconnected; survey-weighted, inverse-variance pooled across contributing entry cohorts. The weighted 3-year cumulative incidence of the composite end point was 25.4% (offline) vs 12.9% (connected non-user) in the round-11 cohort.		

**Table 3:** Hazard ratios for the digital-integration gradient and the hard end points, by adjustment model and analytic method.

Adjustment model	Stacked model (primary)	R11	R12	R13	Meta-analytic (I-squared)
**Composite end point - offline vs non-disconnected**					
M0 (structural terms only)	2.38 (2.00–2.83)	2.20 (1.77–2.74)	2.48 (1.84–3.36)	2.71 (2.01–3.65)	2.39 (2.06–2.79); 0%
M1 (+demographic)	1.78 (1.44–2.20)	1.59 (1.21–2.08)	1.92 (1.35–2.73)	2.12 (1.50–2.98)	1.81 (1.51–2.17); 0%
M2 (+ socioeconomic)	1.61 (1.29–2.00)	1.43 (1.10–1.85)	1.72 (1.19–2.46)	1.94 (1.36–2.78)	1.62 (1.35–1.94); 0%
M3 (+ health/frailty) - primary	1.39 (1.12–1.73)	1.25 (0.95–1.65)	1.50 (1.04–2.18)	1.57 (1.10–2.24)	1.40 (1.16–1.68); 0%
M4 (+ geriatric vulnerability)	1.25 (1.00–1.56)	1.09 (0.82–1.45)	1.40 (0.96–2.04)	1.36 (0.92–2.04)	1.23 (1.01–1.50); 0%
**All-cause mortality - offline vs non-disconnected**					
M0 (structural terms only)	2.66 (2.18–3.24)	2.58 (1.99–3.33)	2.81 (2.05–3.87)	2.59 (1.80–3.74)	2.65 (2.22–3.16); 0%
M1 (+demographic)	1.93 (1.50–2.48)	1.70 (1.20–2.40)	2.29 (1.52–3.43)	1.95 (1.31–2.89)	1.93 (1.55–2.41); 0%
M2 (+ socioeconomic)	1.77 (1.37–2.29)	1.55 (1.10–2.19)	2.09 (1.38–3.16)	1.79 (1.20–2.67)	1.76 (1.41–2.20); 0%
M3 (+ health/frailty) - primary	1.50 (1.16–1.94)	1.32 (0.92–1.89)	1.81 (1.19–2.75)	1.41 (0.96–2.09)	1.48 (1.18–1.85); 0%
M4 (+ geriatric vulnerability)	1.33 (1.03–1.73)	1.14 (0.79–1.65)	1.71 (1.11–2.62)	1.18 (0.76–1.84)	1.30 (1.03–1.65); 10%
**Secondary contrast - telehealth user vs connected non-user (M3)**					
Composite end point	0.99 (0.79–1.24)	1.23 (0.95–1.60)	0.90 (0.69–1.17)	0.73 (0.47–1.13)	1.00 (0.85–1.18); 61%
All-cause mortality	0.99 (0.75–1.30)	1.03 (0.77–1.39)	1.18 (0.86–1.62)	0.66 (0.41–1.05)	1.00 (0.82–1.22); 52%

*Note*. Hazard ratios (95% CI) from survey-weighted discrete-time survival models. The stacked model (entry-cohort fixed effects, participant-level cluster-robust variance) is the primary result; per-cohort and meta-analytic estimates are a consistency check. I-squared = between-cohort heterogeneity. M1 adds age, sex, race/ethnicity, education; M2 adds Medicaid, living alone, marital status; M3 adds comorbidity count, self-rated health, dementia, established cardiovascular disease; M4 adds ADL help, going-outside frequency, depression, self-rated memory, household income.

**Table 4:** Quantitative bias and robustness analyses for the fully adjusted offline-versus-non-disconnected associations.

Analysis	Result
E-values (minimum unmeasured confounding to explain away the association)	Point estimate; confidence-limit value
Composite end point, M3 (HR 1.39)	2.12; 1.47
All-cause mortality, M3 (HR 1.50)	2.36; 1.58
Composite end point, M4 (HR 1.25)	1.80; 1.06
All-cause mortality, M4 (HR 1.33)	2.00; 1.20
**Re-estimation under alternative analytic choices**	
Multiple imputation (m = 10), composite end point	HR 1.40 (1.14–1.73)
Multiple imputation (m = 10), all-cause mortality	HR 1.53 (1.20–1.96)
Residential-care transition, cause-specific model	HR 1.24 (0.84–1.82)
Residential-care transition, Fine-Gray subdistribution	HR 1.18 (0.80–1.74)
**Reverse causation and proxy response (composite end point)**	
Excluding first-interval events, stacked model	HR 1.21 (0.88–1.66)
Excluding first-interval events, R11 / R12	HR 1.01 (0.76–1.33) / 1.57 (0.90–2.75)
Restricting to self-respondents, stacked model	HR 1.34 (1.06–1.69)
Restricting to self-respondents, R11 / R12 / R13	HR 1.23 (0.93–1.64) / 1.43 (0.97–2.11) / 1.46 (0.99–2.14)
**Loss to follow-up (composite end point)**	
Loss to follow-up, offline vs non-disconnected	14.0% vs 11.3% (weighted)
Extreme-scenario bounds on the stacked M3 estimate	HR 1.07 to 1.85

*Note*. The E-value is the minimum strength of association, on the risk-ratio scale, that an unmeasured confounder would need with both exposure and outcome to fully explain the association. HRs reaching or crossing 1 indicate that reverse causation and residual confounding cannot be excluded. The round-13 first-interval-exclusion analysis is omitted because the ~ 1-year window leaves too few residual events.

## Data Availability

NHATS public-use data are available from the official NHATS website (https://nhats.org) upon registration. The analysis code is available from the corresponding author on reasonable request and will be deposited in an open repository on publication. Derived analytic datasets can be reconstructed from the public-use files using the analysis code.

## Abbreviations

ADL: activity of daily living
CI: confidence interval
CVD: cardiovascular disease
HR: hazard ratio
NHATS: National Health and Aging Trends Study
PHQ-2: Patient Health Questionnaire-2
RECORD: REporting of studies Conducted using Observational Routinely collected health Data
STROBE: Strengthening the Reporting of Observational Studies in Epidemiology
